# Genotoxic and Chemopreventive Effects of* Vochysia divergens* Leaves (Pantanal, Brazil)

**DOI:** 10.1155/2018/6596142

**Published:** 2018-09-19

**Authors:** Pollyanna Francielli de Oliveira, Suzana Amorim Mendes, Nathália Oliveira Acésio, Luis Claudio Kellner Filho, Leticia Pereira Pimenta, Kátia Aparecida Siqueira, Marcos Antônio Soares, Ana Helena Januário, Denise Crispim Tavares

**Affiliations:** ^1^Universidade de Franca, Avenida Dr. Armando Salles de Oliveira, 201 Parque Universitário, 14404-600 Franca, SP, Brazil; ^2^Universidade Federal de Alfenas, Rua Gabriel Monteiro da Silva, 700 Centro, 37130-000 Alfenas, MG, Brazil; ^3^Universidade Federal de Mato Grosso, Avenida Fernando Corrêa da Costa, 2367 Boa Esperança, 78060-900 Cuiabá, MT, Brazil

## Abstract

The medicinal plant* Vochysia divergens* is a colonizing tree species of the Pantanal, a unique and little explored wetland region in Brazil. This species is used in folk medicine as syrups and teas to treat respiratory infections, digestive disorders, asthma, scarring, and skin diseases. The objectives of this study were to evaluate the antioxidant, cytotoxic, and genotoxic potential of the ethanolic extract of* Vochysia divergens* leaves (VdE), as well as the influence of VdE and its major component (the flavone 3′,5-dimethoxy luteolin-7-*O*-*β*-glucopyranoside; 3′5 DL) on MMS-induced genotoxicity. The extract significantly reduced the viability of V79 cells in the colorimetric XTT assay at concentrations ≥ 39 *μ*g/mL. A significant increase in micronucleus frequencies was observed in V79 cell cultures treated with VdE concentrations of 160 and 320 *μ*g/mL. However, animals treated with the tested doses of VdE (500, 1000, and 2000 mg/kg b.w.) exhibited frequencies that did not differ significantly from those of the negative control group, indicating the absence of genotoxicity. The results also showed that VdE was effective in reducing MMS-induced genotoxicity at concentrations of 20, 40, and 80 *μ*g/mL in the* in vitro* test system and at a dose of 15 mg/kg b.w. in the* in vivo* test system. Its major component 3′5 DL exerted no protective effect, suggesting that it is not responsible for the effect of the extract. The results of the 2,2-diphenyl-1-picrylhydrazyl (DPPH) assay showed that VdE was able to scavenge 92.6% of free radicals. In conclusion, the results suggest that the protective effect of VdE may be related, at least in part, to the antioxidant activity of its chemical constituents.

## 1. Introduction

The use of medicinal products derived from plants has increased considerably over the last three decades, with about 80% of people using these products for primary health care [1]. The Brazilian Cerrado, located in the central region of the country, is one of the most extensive biomes (204 million hectares) and is considered the richest tropical grassland in the world in terms of biodiversity and the second largest biome in South America [2]. It is estimated that only 30% of this biodiversity is reasonably known [3]. Plants endemic to the Cerrado have been receiving increased attention as a source of bioactive compounds, especially phenolic compounds [4], substances with known antioxidant, chemopreventive, cytoprotective, antimutagenic, antiestrogenic, and antiangiogenic activities [5].

*Vochysia divergens* Pohl (Vochysiaceae), commonly known as Cambará, is native to the Amazon Basin. As settlers of the wetlands of the Brazilian Pantanal, this species is considered an invasive plant that is widely tolerant to seasonal variations in hydrological conditions and is therefore resistant to the dry season as well as to the seasonal floods that occur during the rainy season in the Pantanal [6]. The plant is used in folk medicine to treat respiratory infections, digestive disorders, and asthma. There are also reports of its use for wound healing and treatment of skin diseases. The main nutritional reserves of its seeds are proteins, oils, and few carbohydrates [7].

The extracts of the stem bark of* V. divergens* and some compounds isolated from this plant, beta-sitosterol, betulinic acid, and sericic acid, have been evaluated for antibacterial activity. Sericic acid was the most active compound, a finding that may explain in part the popular use of this plant for the treatment of infectious diseases [8]. Another study identified the schistosomicidal potential of the ethanolic extract of* V. divergens* leaves and isolated flavones [9]. However, few studies have explored* V. divergens*. Considering the importance of this species in folk medicine, we performed a toxicogenetic evaluation of the ethanolic extract of* V. divergens *leaves (VdE) and of its major component, the flavone 3′5-dimethoxy luteolin-7-*O*-*β*-glucopyranoside (3′5 DL), and evaluated their effects on genomic stability and oxidative stress.

## 2. Material and Methods

### 2.1. Plant Material

*Vochysia divergens* was collected in October 2012 in the Pantanal region of Mato Grosso (S16°35′22,90^″^ and W56°47′83,40^″^). A voucher specimen was deposited in the Herbarium of the Federal University of Mato Grosso (UFMT), Brazil (UFMT 39559).

### 2.2. Extract Preparation and Flavone Isolation and Quantification

The leaves of* V. divergens* (1.27 kg) were air-dried, powdered, and exhaustively extracted by maceration in EtOH. These procedures were performed at room temperature. The solution was then filtered and the solvent was removed under reduced pressure, yielding the crude extract (82.83 g VdE). Part of the extract obtained (3 g) was purified over a Sephadex LH-20® column and eluted with methanol to afford seven fractions. Reverse-phase ODS chromatography was performed with Fraction 3 (80 mg), which was purified by preparative RPHPLC [CH3OH-H2O-CH3COOH (50:49.9:0.1, v/v/v)] to yield 3′5 DL (10 mg) [9]. The flavone was quantified in VdE by HPLC-DAD according to Pimenta et al. [10].

### 2.3. In Vitro Test System

#### 2.3.1. Cell Line and Culture Conditions

Chinese hamster lung fibroblasts (V79) were maintained as monolayers in plastic culture flasks (25 cm^2^) in HAM-F10 and DMEM medium (1:1; Sigma-Aldrich Co., St. Louis, MO, USA) supplemented with 10% fetal bovine serum (Nutricell, Campinas, SP, Brazil), antibiotics (0.01 mg/mL streptomycin, CAS:3810-74-0, and 0.005 mg/mL penicillin, CAS:113-98-4; Sigma-Aldrich Co., St. Louis, MO, USA), and 2.38 mg/mL Hepes (CAS:7365-45-8; Sigma-Aldrich Co., St. Louis, MO, USA) at 37°C in a BOD-type chamber (Model: 347CD, FANEM Ltd., São Paulo, SP, Brazil). Protocols were performed in triplicate using cells with a mean cell cycle of 12 hours between the 4th and 12th passage.

#### 2.3.2. Colorimetric XTT Assay

The cytotoxic effects of VdE were determined by monitoring the growth of V79 cells using the Cell Proliferation Kit from Roche Life Science (Indianapolis, IN, USA) after 24 h of incubation. For this purpose, 10^4^ cells were seeded in 96-well plates containing 100 *μ*L HAM-F10 + DMEM medium supplemented with 10% fetal bovine serum. The microplates were incubated in a CO_2_ incubator (Model: MCO18AC, SANYO Electric Co., Ltd., Osaka, Japan) at 37°C. After 24 h, the cells were treated with VdE previously dissolved in 1% dimethyl sulfoxide (DMSO; Sigma-Aldrich, St. Louis, MO, USA) at concentrations ranging from 2.40 to 5,000 *μ*g/mL. Negative (no treatment), solvent (1% DMSO), and positive (DMSO 25%) controls were included. The cells were incubated for 24 h and washed with phosphate-buffered saline (PBS). Each plate received 100 *μ*L HAM-F10 medium without phenol red plus 25 *μ*L XTT. Absorbance of the samples was measured after 17 h in a microplate reader (ASYS-UVM 340/MikroWin 2000, Biochrom, Holliston, MA, USA) at a wavelength of 450 nm and a reference length of 620 nm.

#### 2.3.3. Experimental Design

Concentrations of VdE of 20, 40, 80, 160, and 320 *μ*g/mL were used for genotoxicity assessment. This choice was based on the XTT assay using the criterion of cytotoxicity. Concentrations of 20, 40, and 80 *μ*g/mL were used to study the influence of VdE on the genotoxicity induced by the mutagenic agent methyl methanesulfonate (MMS, 44 *μ*g/mL; Sigma-Aldrich, St. Louis, MO, USA). The concentrations of 3′5 DL were selected based on the proportion of this compound in VdE, corresponding to 0.77%. Thus, the cell cultures were treated with 0.616 *μ*g/mL 3′5 DL for genotoxicity evaluation. The different concentrations of the compound (0.154, 0.308, and 0.616 *μ*g/mL) were combined with MMS for the assessment of its influence on genomic stability. Negative (no treatment), solvent (DMSO 1%), and positive (MMS, 44 *μ*g/mL) controls were included.

#### 2.3.4. Micronucleus Test

A total of 500,000 V79 cells were seeded in a culture flask (25 cm^2^) with 5 mL HAM-F10 + DMEM medium and incubated for 25 h. The cells were then washed with PBS and treated for 3 h with different concentrations of* V. divergens* and the controls in culture medium without fetal bovine serum. The cells were washed twice with PBS and medium containing cytochalasin-B (3 *μ*g/mL; Sigma-Aldrich, St. Louis, MO, USA) and fetal bovine serum was added. The cultures were incubated for 17 h. For micronucleus analysis as described by Fenech [11], 3,000 binucleated cells were analyzed per treatment (1,000 cells/treatment/repetition). The cytotoxicity of the treatments was measured by the nuclear division index (NDI) after the analysis of 1,500 cells (500 cells/repetition). Cells with well-preserved cytoplasm containing 1 to 4 nuclei were scored. The NDI was calculated according to Eastmond and Tucker [12] using the following formula:

(1) $$\begin{array}{c} NDI=\frac{\left[M1+2\left(M2\right)+3\left(M3\right)+4\left(M4\right)\right]}{N} \end{array}$$

where* M1* to* M4* are the number of cells with 1, 2, 3, and 4 nuclei, respectively, and *N* is the total number of viable cells. In addition, the cytotoxicity index (CI) was calculated as proposed by Kirsch-Volders et al. [13]:

(2) $$\begin{array}{c} CI=100-100\left[\;NDI_{T}-\frac{1}{{NDI}_{C}}-1\;\right] \end{array}$$

where *T* represents the different treatments with VdE or 3′5 DL and *C* represents the negative control.

### 2.4. In Vivo Test System

#### 2.4.1. Animals

Male Swiss mice (*Mus musculus*) weighing approximately 25 g were supplied by the Animal House of the School of Pharmaceutical Sciences, University of São Paulo, Ribeirão Preto (SP, Brazil). The animals were kept in plastic boxes in an experimental room with controlled conditions of temperature (23 ± 2°C) and humidity (50 ± 10%) under a 12-h light-dark cycle, with free access to regular laboratory chow and tap water. The study protocol was approved by the Animal Use Ethics Committee of the University of Franca (Approval No. 017/10).

#### 2.4.2. Treatments

Male Swiss mice were divided into groups of six animals each. Considering that the highest dose permitted for* in vivo* genotoxicity testing is 2,000 mg/kg body weight (b.w.) [14], the animals received VdE at doses of 500, 1,000, and 2,000 mg/kg b.w. for genotoxicity assessment. The lower doses (15, 30, and 60 mg/kg b.w.) were combined with MMS (40 mg/kg b.w.) to evaluate the influence of the extract on genotoxicity induced by the mutagen. The extract was dissolved in DMSO (5%) and administered to the animals by gavage in a single dose (0.5 mL/animal). Negative (without treatment), positive (MMS, 40 mg/kg b.w., intraperitoneally), and solvent (5% DMSO) controls were also administered. Bone marrow samples were collected 24 h after treatment.

#### 2.4.3. Micronucleus Test

The bone marrow micronucleus assay was performed according to OECD 474 [14]. The slides were stained with 10% Giemsa in Sorensen's buffer (pH 6.8). The frequency of micronucleated polychromatic erythrocytes (MNPCEs) was determined by analyzing 2,000 polychromatic erythrocytes (PCEs) per animal by light microscopy under oil immersion and 400 erythrocytes per animal were scored to evaluate cytotoxicity (PCE/PCE + NCE [normochromatic erythrocytes]).

### 2.5. DPPH Radical Activity

The free radical scavenging activity of VdE was assessed by the DPPH assay (2,2-diphenyl-1-picrylhydrazyl; CAS 1898–66-4, Sigma-Aldrich, St. Louis, MO, USA). In this assay, antioxidants react with the stable DPPH radical and convert it to 1,1-diphenyl-2-picryl-hydrazine. This capacity is measured by a decrease in absorbance in 96-well plates. VdE and the positive control (gallic acid, 98% purity; CAS149–91-7, Sigma-Aldrich) were individually added to 67.6 mM DPPH in methanol at concentrations of 1.67–66.7 *μ*g/mL methanol and the mixture was incubated for 30 min at 25°C in the dark. Remaining DPPH was determined colorimetrically at 517 nm by comparison with methanol (negative control) in a microplate reader (ASYS-UVM 340/MikroWin 2000, Biochrom, Holliston, MA, USA). The free radical scavenging activity is expressed as percent mean values obtained in triplicate using the following formula:

(3) $$\begin{array}{c} \%\text{ Scavenging}=\left[\;1-\left(\;\frac{A_{\text{sample}}}{A}\;\right)\times 100\;\right], \end{array}$$

where *A* is the absorbance without sample (only solvent and free radical) and *A*_sample_ is the absorbance obtained with the crude extract or gallic acid [15].

### 2.6. Statistical Analysis

The data were analyzed by analysis of variance for completely randomized experiments, with calculation of P values. In cases in which* P* < 0.05, treatment means were compared by the Tukey test and the minimum significant difference was calculated for *α*=0.05. All statistical analyses were performed using the GraphPad Prism 5.0 program (GraphPad Software, San Diego, CA, USA).

## 3. Results

### 3.1. Quantitative HPLC-DAD Analysis

**Figure 1:**
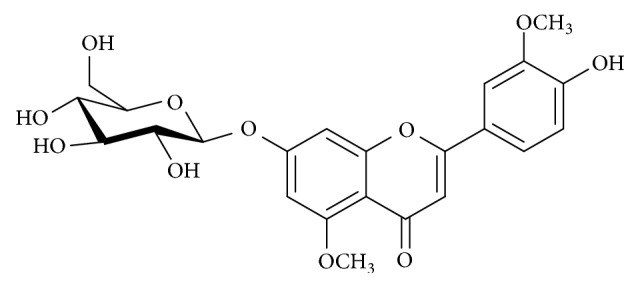
Chemical structure of 3′,5-dimethoxy luteolin-7-*O*-*β*-glucopyranoside.

**Figure 2:**
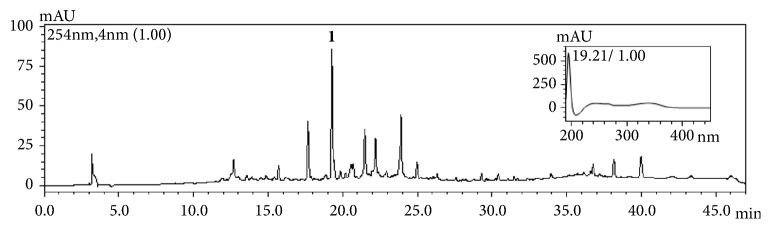
HPLC-DAD chromatogram of the ethanolic extract of* Vochysia divergens*. Chromatographic conditions: CH_3_OH-H_2_O-CH_3_COOH (50:49.9:0.1, v/v/v) gradient from 5 to 100% methanol for 30 min, followed by elution with 100% methanol for 10 min. The injection volume was 20 *μ*L and the detection wavelength was 254 nm.

The content of the flavonoid 3′5 DL (Figure 1, Rt = 19.21 min) in the ethanolic extract determined by HPLC-DAD (Figure 2) was 15.48 ± 0.013 mg/mL, corresponding to 7.74 mg/g dry weight.

### 3.2. Colorimetric XTT Assay

**Figure 3:**
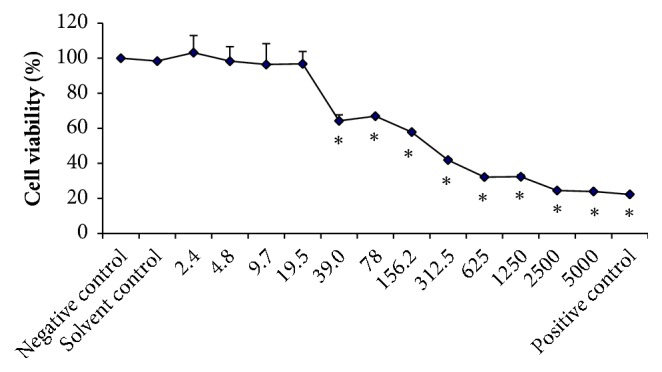
Effects of 24-h treatment with the indicated concentrations of the ethanolic extract of* Vochysia divergens* leaves on V79 cell viability evaluated by the XTT assay. Values are expressed as the mean ± SD. The IC_50_ value was 101.0 ± 8.9 *μ*g/mL. Negative control: no treatment; solvent control: 1% dimethyl sulfoxide (DMSO); positive control: 25% DMSO. *∗*Significantly different compared to the negative control (*P* <0.05).

Figure 3 shows the cytotoxicity of VdE evaluated by the colorimetric XTT assay in V79 cells at concentrations ranging from 2.4 to 5,000 *μ*g/mL. Significant differences were found for concentrations equal to or higher than 39 *μ*g/mL when compared to the negative control, demonstrating a cytotoxic effect (Figure 3).

### 3.3. In Vitro Micronucleus Test

No significant differences in micronucleus frequencies were observed between cultures treated with 20, 40, or 80 *μ*g/mL VdE and the negative control. On the other hand, there was a significant increase of micronucleus frequencies in cultures treated with VdE at concentrations of 160 and 320 *μ*g/mL when compared to the negative control group. Combined treatment resulted in significantly lower micronucleus frequencies in all cultures treated with VdE and MMS compared to those treated with MMS alone. These results indicate the lack of a dose-response relationship for genotoxicity induced by MMS.

The NDI was significantly lower in cultures treated with 320 *μ*g/mL of VdE when compared to the negative control, with a CI of 35.61%. No significant differences in NDI were observed between the other cultures treated with VdE, alone or combined with MMS, and untreated cultures, indicating the absence of cytotoxicity (Table 1).

Evaluation of the major component of the extract, 3′5 DL, revealed the absence of genotoxicity and cytotoxicity at the highest concentration tested (0.606 *μ*g/mL). No differences in micronucleus frequencies were observed between cultures treated with 3′5 DL plus MMS and those treated with MMS alone (Table 1).

### 3.4. In Vivo Micronucleus Test

The frequencies of MNPCEs in Swiss mouse bone marrow treated with different doses of VdE alone or combined with MMS are shown in Table 2. There was no significant difference in the frequencies between animals treated with the tested doses of VdE (500, 1,000, and 2,000 mg/kg b.w.) and the negative and solvent control groups, indicating the absence of genotoxicity. In addition, the oral administration of 15 mg/kg b.w. of VdE concomitantly with the injection of MMS led to a significant reduction in the frequency of MNPCEs when compared to the group treated with MMS alone. Evaluation of cytotoxicity revealed no significant differences in the ratios of PCE/total erythrocytes between the different treatments and the negative control, indicating the absence of cytotoxicity.

### 3.5. DPPH Scavenging Activity

**Figure 4:**
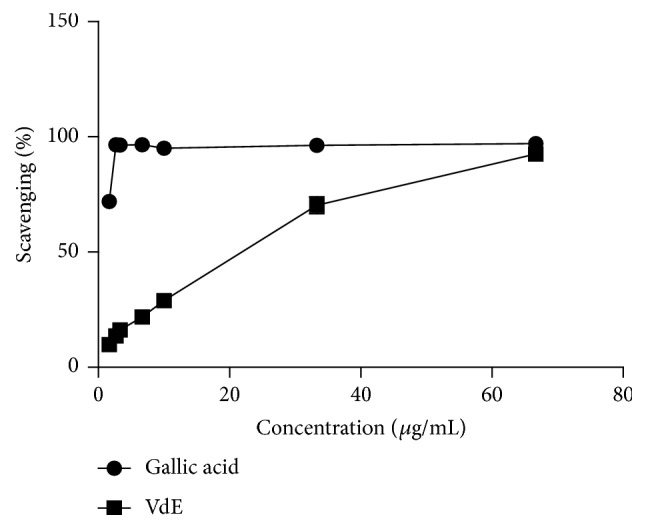
Percentage of free radical-scavenging activity of different concentrations of the ethanolic extract of* Vochysia divergens* leaves (VdE) in the DPPH assay.

Figure 4 shows the mean sequestration frequency of the DPPH radical obtained for the different concentrations of VdE used to evaluate its possible antioxidant activity. The results demonstrate antioxidant capacity of the extract, with a dose-dependent response and maximum inhibition of 92.6% at the highest concentration.

## 4. Discussion

The present results showed that VdE exerted genotoxic activity at the highest concentrations tested in the* in vitro* test system. The VdE presents flavones in its chemical composition, being the flavone 3′5 DL its major component [9, 10]. Polyphenolic compounds, as flavones, can act as prooxidants in some in vitro systems [16]. Some polyphenols may have carcinogenic or genotoxic effects at high doses or concentrations. It is possible that the genotoxic effects observed* in vitro* may be attributable to the high concentration used, at which polyphenols may become prooxidants [17, 18].

However, when the extract was evaluated in the* in vivo* test, no genotoxicity was observed. These divergences in the results between the two test systems may be explained by factors such as metabolism, pharmacokinetics, and DNA repair processes, which are active in the* in vivo* test system and contribute to the responses observed.

Analysis of the influence of VdE on the genotoxicity induced by MMS revealed a chemopreventive effect of the extract. The major component present in VdE, 3′5 DL, was also evaluated* in vitro* to correlate the data obtained with the possible activity of its chemical constituents. However, no protective effect of the flavone on MMS-induced genotoxicity was found. This finding suggests that the chemopreventive activity of VdE may be due to the synergistic effect of its chemical constituents.

Our study demonstrated that VdE exhibits the characteristics of Janus compounds, i.e., substances that behave as genotoxic or antigenotoxic agent at different concentrations depending on the conditions used. The extract was genotoxic at the highest concentrations tested, whereas it exerted a chemopreventive effect in the* in vitro* test system at the lower concentrations. Several molecular mechanisms underlying the Janus effect have been postulated; however, the specific induction and consequent saturation of certain enzymes of an antimutagenic system such as DNA repair seem to be more likely [16].

Chemical studies of the genus* Vochysia* (Vochysiaceae) reported the presence of ellagic acid, physcion, 2,6-dimethoxy,4-benzoquinone [17], a pyrrolidinoflavone [18], 3-O-*B*-D-glucopyranosyl-*β*-sitosterol, and two dicarboxylated triterpenes (bartogenic acid and vismiaefolic acid) [19]. Pimenta et al. [9, 10] demonstrated flavones as constituents of* Vochysia* extracts. Flavones are a class of flavonoids that are a subject of increasing interest because of their biological activities* in vitro* and* in vivo*, especially their antioxidant activity. Flavones from plants are typically bound to sugar units such as glycosides, especially 7-*O*-glycosides [20], and may also contain acetyl or malonyl moieties. Flavone* O*-glycosides are composed of the aglycone moiety and one or more sugars attached through a *β*-linkage [21].

MMS is an SN2 class type mutagenic agent that causes N-alkylation of purines [22]. Additionally, MMS is known to facilitate the formation of adducts such as* N7*-methylguanine (*N7*MeG),* N3*-methylguanine (*N3*MeG), and* N3*-methyladenine (*N3*MeA), as well as crosslinks expressed as base substitution mutations. Although DNA adducts do not directly block replication, they produce apurinic sites, with consequent breaks in the double strands [23] that are repaired by base excision, the main defense mechanism against SN2 agents [24]. Regarding the mechanism of action underlying MMS-induced genotoxicity, VdE may act as a chemoprotector by competing with DNA as a target for alkylation, reducing MMS-induced genotoxic damage.

Alkylating agents have been shown to deplete the enzyme glutathione S-transferase in mammalian cells, leading to oxidative stress as a byproduct of normal cellular function which can compromise cellular antioxidant defenses [25]. Considering that alkylating agents may play a role in the generation of reactive oxygen species, the antioxidant compounds present in VdE may be responsible for the reduction in the alkylation damage induced by MMS. The antioxidant activity of VdE was demonstrated by the DPPH reduction assay in the present study. This assay is widely used as a model system of free radical scavenging activity in plants.

Research on natural sources of antioxidant compounds is being conducted because of their importance for preventing the onset of oxidative reactions. Antioxidants act by delaying or preventing the oxidation of substrates involved in oxidative processes, inhibiting the formation of free radicals. As observed for VdE, phytochemical studies have demonstrated the presence of triterpenoids, steroids, and polyphenols in the genus* Vochysia* and in the family Vochysiaceae. In addition, polyphenolic compounds such as ellagic acid derivatives are considered chemical markers of the family Vochysiaceae [7].

Erratic absorption by the cell membrane and the consequent inconstant bioavailability of the compounds in the cell explain the absence of a significant dose-dependent effect of VdE. In addition, the assessment of dose effects is complicated by the fact that many chemoprotective compounds act simultaneously at different levels of protection [26]. Thus, the lack of observation of a dose-response relationship might be attributed to the activation of different mechanisms depending on the dose of the extract used.

## 5. Conclusions

The present results showed a genotoxic effect of VdE only at the higher concentrations tested (160 and 320 *μ*g/mL), and no genotoxicity was observed in the* in vivo* test system. On the other hand, VdE was effective in reducing the genotoxicity induced by MMS. The major component 3′5 DL exerted no protective effect, indicating that it is not responsible for the effect of the extract. The results suggest that the protective effect of VdE may be related to the antioxidant activity of its chemical constituents.

## Figures and Tables

**Table 1:** Mean micronucleus frequency (MN), nuclear division index (NDI), and cytotoxicity index (CI) obtained for V79 cells treated with VdE and its component, 3′5 DL, alone and combined with MMS.

**Treatment** **(**μ**g/mL)**	**MN frequency ** ^**a**, **b**^	**NDI ** ^**a**, **c**^	**CI (**%**)**
Negative control	7.33 ± 1.53	1.73 ± 0.05	-
DMSO	10.33 ± 3.06	1.71 ± 0.03	2.73
MMS	44.33 ± 2.51 ^d^	1.68 ± 0.01	11.68
DMSO + MMS	41.33 ± 4.04 ^d^	1.79 ± 0.03	ND
VdE			
20	8.67 ± 1.15	1.73± 0.08	0.0
40	10.67 ± 1.53	1.74 ± 0.01	ND
80	11.67 ± 1.53	1.75 ± 0.08	ND
160	17.33 ± 1.15^d^	1.68 ± 0.03	6.84
320	19.67 ± 2.08 ^d^	1.47 ± 0.11^c^	35.61
20 + MMS	19.00 ± 3.00 ^d, e^	1.82 ± 0.07	ND
40 + MMS	23.33 ± 3.51 ^d, e^	1.85 ± 0.09	ND
80 + MMS	25.66 ± 3.21 ^d, e^	1.79 ± 0.17	ND
3′5 DL			
0.616	10.66 ± 2.08	1.71 ± 0.05	7.79
0.154 + MMS	50.00 ± 8.64 ^d^	1.76 ± 0.01	1.29
0.308 + MMS	53.00 ± 6.58 ^d^	1.67 ± 0.09	12.98
0.616 + MMS	38.66 ± 2.51 ^d^	1.57 ± 0.14	25.90

VdE: ethanolic extract of* Vochysia divergens* leaves; 3′5 DL: 3′,5-dimethoxy-luteolin-7-*O*-*β*-glucopyranoside; DMSO: dimethyl sulfoxide (5%); MMS: methyl methanesulfonate (44 *μ*g/mL): ND: not determined. The NDI of the treated group is higher than the NDI of the negative control group. The concentrations of 3′5 DL used were established based on the tested concentrations of VdE (20, 40, and 80 *μ*g/mL), corresponding to the proportion of the compound in the extract, which is 0.77%.  ^a^Values are the mean ± standard deviation.  ^b^A total of 3,000 binucleated cells were analyzed per treatment group.  ^c^A total of 1,500 cells were analyzed per treatment group.  ^d^Significantly different from the negative control group (*P* < 0.05).  ^e^Significantly different from the MMS group (*P* < 0.05).

**Table 2:** Frequencies of micronucleated polychromatic erythrocytes (MNPCEs) and PCE/PCE+NCE ratio in Swiss mouse bone marrow treated with VdE and/or MMS and their respective controls.

**Treatment** **(mg/kg b.w.)**	**MNPCEs ** ^**a**, **c**^	**PCE/PCE + NCE ** ^**a**, **b**^
Control	6.33 *±* 1.75	0.63 *±* 0.13
DMSO	6.33 *±* 2.34	0.63 *±* 0.08
500	4.33 *±* 1.96	0,.66 *±* 0.09
1000	4.16 *±* 1.47	0.72 *±* 0.08
2000	7.83 *±* 2.78	0.60 *±* 0.05
MMS	41.50 *±* 9.13 ^d^	0.61 *±* 0.10
DMSO + MMS	48.00 *±* 9.40 ^d^	0.61 *±* 0.06
15 + MMS	22.83 *±* 2.48 ^d, e^	0.63 *±* 0.05
30 + MMS	42.60 *±* 6.43 ^d^	0.55 *±* 0.12
60 + MMS	47.00 *±* 5.17 ^d^	0.62 *±* 0.05

VdE: ethanolic extract of* Vochysia divergens* leaves; PCE: polychromatic erythrocytes; NCE: normochromatic erythrocytes; DMSO: dimethyl sulfoxide (5%); MMS: methyl methanesulfonate (40 mg/kg b.w.).  ^a^Values are the mean ± standard deviation.  ^b^A total of 400 erythrocytes were analyzed per animal, corresponding to 2,400 cells per treatment.  ^c^A total of 2,000 PCEs were analyzed per animal, corresponding to 12,000 cells per treatment.  ^d^Significantly different from control (*P *< 0.05).  ^e^Significantly different from the MMS group (*P *< 0.05).

## Data Availability

The readers can access the data that support the conclusions of the present study through the tables and figures presented.
